# Unsupervised genome-wide recognition of local relationship patterns

**DOI:** 10.1186/1471-2164-14-347

**Published:** 2013-05-24

**Authors:** Neda Zamani, Pamela Russell, Henrik Lantz, Marc P Hoeppner, Jennifer RS Meadows, Nagarjun Vijay, Evan Mauceli, Federica di Palma, Kerstin Lindblad-Toh, Patric Jern, Manfred G Grabherr

**Affiliations:** 1 Science for Life Laboratory, Department of Medical Biochemistry and Microbiology, Uppsala University, Uppsala, Sweden; 2 Broad Institute of MIT and Harvard, Cambridge, MA, USA; 3 Department of Ecology and Genetics, Evolutionary Biology Centre, Uppsala University, Uppsala, Sweden; 4 Boston Children’s Hospital, Boston, MA, USA

## Abstract

**Background:**

Phenomena such as incomplete lineage sorting, horizontal gene transfer, gene duplication and subsequent sub- and neo-functionalisation can result in distinct local phylogenetic relationships that are discordant with species phylogeny. In order to assess the possible biological roles for these subdivisions, they must first be identified and characterised, preferably on a large scale and in an automated fashion.

**Results:**

We developed *Saguaro*, a combination of a Hidden Markov Model (HMM) and a Self Organising Map (SOM), to characterise local phylogenetic relationships among aligned sequences using *cacti*, matrices of pair-wise distance measures. While the HMM determines the genomic boundaries from aligned sequences, the SOM hypothesises new cacti in an unsupervised and iterative fashion based on the regions that were modelled least well by existing cacti. After testing the software on simulated data, we demonstrate the utility of *Saguaro* by testing two different data sets: (i) 181 Dengue virus strains, and (ii) 5 primate genomes. *Saguaro* identifies regions under lineage-specific constraint for the first set, and genomic segments that we attribute to incomplete lineage sorting in the second dataset. Intriguingly for the primate data, *Saguaro* also classified an additional ~3% of the genome as most incompatible with the expected species phylogeny. A substantial fraction of these regions was found to overlap genes associated with both the innate and adaptive immune systems.

**Conclusions:**

*Saguaro* detects distinct cacti describing local phylogenetic relationships without requiring any a priori hypotheses. We have successfully demonstrated *Saguaro*’s utility with two contrasting data sets, one containing many members with short sequences (Dengue viral strains: *n* = 181, genome size = 10,700 nt), and the other with few members but complex genomes (related primate species: n = 5, genome size = 3 Gb), suggesting that the software is applicable to a wide variety of experimental populations. *Saguaro* is written in C++, runs on the Linux operating system, and can be downloaded from http://saguarogw.sourceforge.net/.

## Background

The phylogenetic relationship between organisms on a local genomic level does not always directly reflect the history of speciation. This can be due to well-known phenomena such as gene duplication and subsequent sub- and neo-functionalisation (reviewed in [1]), population subdivision and asymmetric gene flow [2], introgression [3], incomplete lineage sorting [4], hybridisation [5], copy number variation [6], and parallel adaptive evolution [7]. Identifying the regions subjected to these processes promises important insights into genome evolution, as we can relate these changes back to their expected biological roles, and in extension, the possible evolutionary pressures that ensured the survival of these regions within the studied population. We previously used a machine-learning algorithm that incorporated a Hidden Markov Model (HMM) [8] and a Self-Organising Map (SOM, a type of artificial neural network) [9], to investigate the genomes of sticklebacks. There, we detected distinct signatures of local phylogenies that are discordant with ancestry, which we could attribute to the effect of parallel adaptive evolution [7]. We now expand the scope of this algorithm and present the software, *Saguaro*.

A number of analysis tools have been developed to measure differences in local phylogenies, including but not limited to Phylo-HMM [10], SiPhy [11], and Coal-HMM [4,12]. While these methods detect changes in phylogenetic tree size and branch lengths, or match local regions with a set of phylogenetic hypotheses, they lack a component to learn hypotheses directly from the data and without supervision. This is a particularly relevant limitation when analysing a large number of genomes, since these methods have no means of detecting patterns that were not anticipated. *Saguaro* fills the gap left by these methods in that it learns from the data without input of any a priori hypotheses. However, it does not provide the biological interpretation of its findings, but instead helps in generating new questions and perspectives.

At any given position in a multiple sequence alignment, the nucleotides in different genomes are either identical with each other, or not. Consequently, this local relationship is best described as a *binary* phylogeny that is built from this single nucleotide site. Wider branching patterns and branch lengths only become apparent as the average of adjacent binary trees, and from those, more meaningful phylogenetic patterns can be inferred. In order to accommodate a phylogeny that can be built up from such binary trees, *Saguaro* is based on the concept of a *cactus*. Given *n* genomes, a cactus is a symmetric matrix of n*n pairwise genome comparisons, where each element describes how different two genomes are relative to all other pairs. Restricting input sites to positions in which a minimum number of genomes differ from the rest normalizes the elements in the matrix, both in terms of phylogenetic branch lengths, as well as the branching itself. The purpose of a cactus is thus to represent segments of the genome in which consecutive input sites, as a whole, best match a particular cactus, without a cactus providing any immediate biological meaning. While the segmentation can be efficiently computed by a HMM, the next challenge is to a priori hypothesise the shape of the cactus that best represents the genomic segments. To achieve this, *Saguaro* utilises a SOM, which is an efficient unsupervised pattern recognition and classification algorithm. SOMs have been used in bioinformatics, including classification of the selectivity of inhibitors [13], image analyses of fungal colonies [14], and transcription factor binding site identification [15]. A feature that distinguishes a SOM from other clustering and classification algorithms is that it models the topology of the input data onto its neurons by reducing the dimensionality of the input space. In this regard, it can be considered a non-linear generalisation of Principal Components Analysis [16], which is a widely used multivariate analysis algorithm to automatically group data points by patterns. The purpose of *Saguaro’s* SOM is to iteratively build up a set of cacti differing in the phylogeny that they describe, so that the local relationship between sequences in each region is well represented by at least one cactus.

Here, we first explain the methods behind *Saguaro*, and continue by presenting results from analyses using two different data sets: (i) many genomes of short lengths: 181 strains of the Dengue virus serotype 3 isolated from different geographical locations over several years and from various outbreaks [17]; and (ii) few, but complex, large genomes: five primates including human, chimpanzee, gorilla, orang-utan, and macaque.

## Implementation

**Figure 1:**
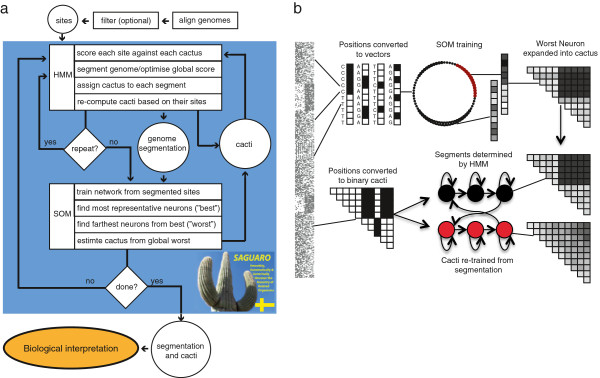
***Saguaro*’s workflow.** (**a**) *Saguaro* takes genome-wide aligned nucleotide sites in genomic order as input. The filtering of non-informative nucleotides is configurable. A Hidden Markov Model (HMM) scores each site against cacti and segments the genome by the best statistical fit. Each cactus is then re-computed by all the sites it represents. This step can be repeated to refine both the segmentation and the cactus. To augment the set of cacti, *Saguaro* trains one Self Organizing Map (SOM) per cactus from all the sites residing in segments represented by this cactus. In each SOM, *Saguaro* then finds the neuron that best represents its sites, and based on that, determines the neuron farthest from that as the worst representative given its sites. *Saguaro* then picks the SOM with the longest distance between the best and worst neurons and hypothesises a new cactus from this worst neuron. This cactus is passed back to the HMM which assigns segments to it. (**b**) Shown is a low-level schematic of how *Saguaro* processes input into output. During the SOM stage, input sites are translated into binary vectors of 1’s (mismatch, black) and 0’s (matches, white), relative to a randomly chosen genome that serves as the ‘reference’. The SOM is then presented with these vectors in random order, so that the continuous vectors contained in the neurons model the input space. The most common input pattern results in the highest density of neurons, whereas patterns not well-modelled by these neurons form their own cluster (shown in red). These neurons are then expanded into a cactus, and added to the HMM’s set. The HMM then re-segments the genome and re-trains the cacti. This process is repeated iteratively in order to build a set of cacti that model different subsets of the genome via their patterns.

*Saguaro*’s basic workflow is shown in Figure 1a. After the genomes have been aligned, *Saguaro* first builds one cactus from all differences found in the entire genome, and then iteratively adds more cacti to refine representations for different subsets of the genome. In each iteration, it scores each nucleotide site against a set of cacti, using the HMM to determine segment boundaries. Then, *Saguaro* re-computes each cactus based on the sites in its segments to further improve the cactus’ representation of its sites. *Saguaro* then trains a SOM for each cactus. This allows the software to further partition the pattern space, identifying genomic regions that are not well modelled by any of the cacti in the current set, and hypothesise additional cacti that are more representative of these regions. Each SOM is trained with randomly chosen sites from regions assigned to its cactus so that the neurons model local patterns from these positions (see section “Self Organising Map”). This subdivision of the input space serves to hypothesise new cacti by examining the shape of the SOM after training.

Figure 1b is a schematic of the inner mechanisms of *Saguaro*. After segmenting the genome into regions, the SOM is presented with random sites from regions assigned to the same cactus. Input sites are transformed into binary vectors where white indicates nucleotide matches and black represent mismatches. The SOM is trained from *binary* vectors into neurons that are represented by *continuous* vectors. As a result, the neurons cluster by frequency of input patterns, with the most prominent pattern forming the tightest cluster with the highest density of data points. *Saguaro* then finds the second-most weighted cluster at a minimum distance from the highest weighted cluster, representing input sites that are most abundant in the input data but least well modelled by the cactus they were assigned to. The data vector from these neurons is then expanded into a cactus and added to the HMM’s set of cacti. In the next round of iterations, the HMM re-segments the input data and re-estimates all cacti. This process is repeated for a set number of iterations, after which the final output is computed as a segmentation of the input sequences into different phylogenetic patterns for further examination and biological interpretation.

### Input and output formats

Input data needs to be converted into *Saguaro*-native binary format. *Saguaro* provides conversion tools for Multiple Alignment Format (MAF), Variant Call Format (VCF), and multi-FASTA format of aligned genomes. Filtering out uninformative sites is configurable and implemented during conversion. At the end, *Saguaro* also computes a local cactus for each individual region.

### Hidden Markov Model (HMM)

The states of the HMM are defined by cacti, applying a flat penalty when transitioning between states and requiring a minimum stay duration of three consecutive nucleotide sites, modelled by three sequential states. Given *n* genomes, for each nucleotide site, we construct the observed matrix *O* of size n*n, which is a binary matrix of 0’s (match) and 1’s (mismatch) based on pairwise comparisons. We next define the scoring scheme *S(H,O)* to compare a cactus *H* to matrix *O*. We can think of a possible nucleotide substitution between genome *i* and *j* (*i* ≠ *j*) as a Poisson process with parameter *H*_*i,j*_ representing a measure for the distance between genome *i* and *j* compared to all other pairwise comparisons. Since the observed number of substitutions *O*_*i,j*_ is either 0 or 1, the likelihood *l*_*i,j*_ of the individual observation *O*_*i,j*_ is:

$$l_{i,j}=\left\{\begin{array}{c} e^{-H_{i,j}}O_{i,j}=0\\ 1-e^{-H_{i,j}}O_{i,j}=1 \end{array}\right)$$

Which can be summarised in one expression as:

$$l_{i,j}=e^{-H_{i,j}}+O_{i,j}-2O_{i,j}\;e^{-H_{i,j}}$$

Assuming independence across all genomes, the likelihood *L(O,H)* of the entire observation *O* is the product of all the individual likelihoods *l*_*i,j*_. This gives:

$$L\left(O,H\right)=\underset{i\neq j}{\prod }\left(e^{-H_{i,j}}+O_{i,j}-2O_{i,j}\;e^{-H_{i,j}}\right)$$

*L(O,H)* is positive as long as *H*_*i,j*_ ≠ 0. We let the final score, *S(H,O)*, be the log of the likelihood score *L(H,O*):

$$S\left(O,H\right)=\underset{H_{i,j}\neq 0}{\sum }\log\left(e^{-H_{i,j}}+O_{i,j}-2O_{i,j}\;e^{-H_{i,j}}\right)$$

If genome *i* or *j* (or both) do not have any information at the given position, we set *O*_*i,j*_ = −1. The score *S(H,O)* is then:

$$S\left(O,H\right)=\underset{\begin{array}{c} H_{i,j}\neq 0\\ O_{i,j}\neq -1 \end{array}}{\sum }\log\left(e^{-H_{i,j}}+O_{i,j}-2O_{i,j}\;e^{-H_{i,j}}\right)$$

Subsequent to each segmentation, we update all cacti by modifying *H* to represent more of the observations indexed by the set *R* ∈ *N*. We minimise the total score *S’* of *H* over all the observations:

$$S^{'}\left(H,R\right)=\underset{r\in R}{\sum }S\left(H,O_{r}\right)$$

Since *S(H, O*_*r*_*)* is the sum of log likelihood scores over all genome pairs, we can optimise each *H*_*i,j*_ individually.

For a single pair of genomes (*i*, *j*), let:

a_0_ = the number of observations in {*O*_*r*_} in which genome *i* and *j* agree

a_1_ = the number of observations in {*O*_*r*_} in which genome *i* and *j* disagree

Undefined observations are not included. We thus maximise the total score over all observations

$$\begin{array}{ll} S{'}_{i,j} & =\underset{r\in R}{\sum }\log\left(e^{-H_{i,j}}+O_{i,j}-2O_{i,j}\;e^{-H_{i,j}}\right)\\ & =-a_{0}H_{i,j}+a_{1}\log\left(1-e^{-H_{i,j}}\right) \end{array}$$

*S’*_*i,j*_ is a differentiable function of *H*_*i,j*_ which attains its maximum at

$$H^{*}{}_{i,j}=\left\{\begin{array}{c} -\log\left(\frac{a_{0}}{a_{0}+a_{1}}\right)\;a_{0}\neq 0\\ \infty \;a_{0}=1\; \end{array}\right)$$

Thus, we update *H* for the data in {*O*_*r*_} by setting:

$$H_{i,j}=\left\{\begin{array}{c} -\log\left(\frac{a_{0}}{a_{0}+a_{1}}\right)a_{0}\neq 0\\ \log\left(a_{1}\right)a_{0}=1\; \end{array}\right)$$

for all pairs (*i*,*j*).

### Self Organizing Map (SOM)

*Saguaro*’s Self Organizing Map (SOM) is organised in a circle. Given the number of genomes *n*, each neuron contains a vector *f* with size *n,* and its elements *f*_*i*_ are initially assigned random values between 0 and 1. To train the neural network, input positions are randomised in order, and each input position is converted into a vector *l* of size *n*, with each element *l*_*i*_ either set to 0 or 1, depending on whether the nucleotides are identical (0) or not (1) compared to one randomly chosen genome that serves as the reference for the site. We then compute the distance between this vector and each vector *f*^*j*^ of neuron *j* as:

$$d_{j}=\min\left(\frac{\sum_{i}{\left(f_{i}^{j}-l_{i}\right)}^{2}}{n},\frac{\sum_{i}{\left(f_{i}^{j}-\left(1-l_{i}\right)\right)}^{2}}{n}\right)$$

Based on this distance measure, we determine the neuron *g* with the shortest distance. All neurons *j* are then updated as:

$$f_{i}^{j'}=f_{i}^{j}\left(1-w\right)+wl_{i}$$

where the weight w is defined as:

$$w=\frac{h}{\min\left({\left(j-g\right)}^{2},{\left(N-j-g\right)}^{2}\right)+1}$$

with *N* being the number of neurons, and *h* monotonically decreasing with the number of processed input sites.

### Parameter choice

*Saguaro* has two main parameters: (i) the penalty applied by the HMM when transitioning between different cacti, and (ii) the number of neurons in the self-organizing map. To investigate parameter sensitivity, we previously applied *Saguaro* to genomic re-sequencing data from the twenty populations of sticklebacks in which we previously identified signatures of adaptive evolution using this method, as well as a hypothesis-driven statistical approach [7]. We ran *Saguaro* on all of chromosome IV with transition penalties of 50, 100, 150, and 200. For values of 50, 100, and 150, *Saguaro* found the signature of adaptive evolution within four iterations, while a transition penalty of 200 required seven iterations, suggesting a drop-off in sensitivity above 150. We next varied the number of SOM neurons, using 200, 400, and 800 respectively. While 400 and 800 neurons yielded identical results over the first five iterations, the use of 200 neurons required one additional iteration before the signature was found, suggesting a drop in sensitivity at this value or lower. After 20 iterations, each run yielded very similar results, suggesting that (apart from using extreme values) the choice of parameters mostly affects runtime, and that the algorithm is robust with regards to parameter settings. Based on the test above and in absence of any training data particular to the data sets, we selected a transition penalty of 150 and 800 SOM neurons for the analyses described here, the same values that were used in the stickleback study.

## Results

### Simulated data

**Figure 2:**
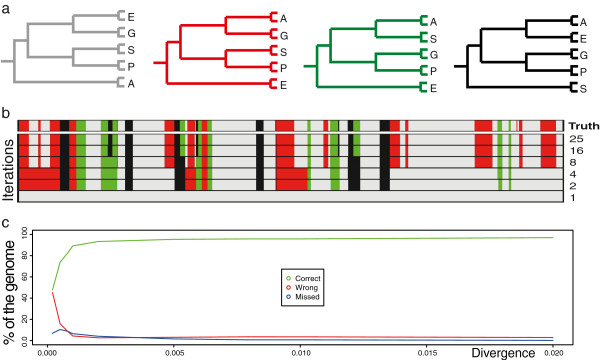
**Simulated data.** (**a**) We simulated the genomes of 10 individuals grouped into 5 populations or species, labelled A, E, G, P and S. We segmented the genomes in blocks of 50 to 1000 nucleotides and assigned different local phylogenies at different frequencies, with the tree depicted in grey covering most of the genome and black the least coverage. (**b**) Shown is the comparison of *Saguaro’s* segmentation with the simulated truth (top). After iteration 16, Saguaro segments the genome correctly according to the simulation, with the exception of one block containing 9 SNPs. (**c**) We varied the divergence between genomes from 0 to 0.2 and computed the fraction of all nucleotides, including invariant sites, that was assigned to regions in which topology agrees with the simulation (green), disagrees (red), or falls in invariant regions at segmentation boundaries (blue).

We first generated a simulated data set, based on a 100 Kb genome. Genomes for 10 individuals were simulated in blocks of random size (50–2000 nucleotides) using the program Dawg [18] version 1.2 by specifying one of four phylogenies (Figure 2a) with mutations at a rate of 0.1-1% per generation. In order to simulate uneven abundance of these phylogenies, we set the probabilities to 0.5, 0.25, 0.125, and 0.0625 to choose these phylogenies, allowing for consecutive blocks of the same phylogeny. Figure 2b shows a visual representation of Saguaro’s output after different numbers of iterations, compared to the “truth” input set for the simulation at the top. For comparison, we computed local phylogenies and coloured the segments according to which simulated phylogeny was most closely matched, as determined by TOPD [19]. As soon as in the second iteration, where only two cacti are available for segmentation, *Saguaro* starts detecting segment boundaries correctly. After 16 iterations, *Saguaro* segments the genome into blocks closely resembling the truth, with the shortest block being 91 bp long and containing 3 SNP's. Only one 170 nucleotide long region with 9 SNPs was not identified correctly (red line in the upper right corner in Figure 2b).

In order to determine sensitivity, we next varied the divergence rates from 0 to 0.02 (Figure 2c). To measure the performance of the segmentation, we computed tree topologies for each segment, and counted the percentage of the genome that was either: (a) assigned to a topology accurately representing the simulation (“correct”); (b) assigned to a topology different from the simulation (“wrong”); and (c) the percentage not assigned to any cactus, i.e. invariant regions between SNPs at the segment boundaries (“missing”). As expected, assignments were more accurate with increasing divergence, starting to level out at around 0.002 (Figure 2c).

### Local pattern variation in Dengue virus phylogeny

Dengue viruses are mosquito-borne single-stranded RNA viruses of the *Flaviviridae* family that infect humans with between 50 to 100 million cases reported every year [20]. Over several years, 181 Dengue virus serotype 3, strains have been collected from various geographic locations in Central and South America (Venezuela, Colombia, Brazil, Puerto Rico, Nicaragua, Caribbean) as well as Asia (Sri Lanka, Thailand) [17]. Schmidt et al. reported that the genome-wide phylogeny is reflective primarily of geographic location, but also of the year of outbreak. The Dengue virus genome size is small at around 10,660 nucleotides, and we thus hypothesise that selection criteria may exert pressure on very localised regions in the virus. To explore this, we first extracted a total of 1260 single nucleotide differences and short insertions and deletions (indels) from multiple sequence alignments [17]. Variants supported by at least three Dengue virus strains were classified as phylogenetically informative. Iterative runs of *Saguaro* produced five different cacti, with cacti 1 to 4 being very similar to each other, but with cactus 5 being distinct. To independently validate whether these cacti describe changes in local phylogeny, we used a pipeline [21] consisting of MUSCLE [22], Gblocks [23], and PhyML [24] to re-align different genomic sequences segmented into cacti directly, and to build a phylogeny based on all nucleotides, including identical sites. A Dengue virus serotype 1 sequence was used as outgroup in the phylogeny.

**Figure 3:**
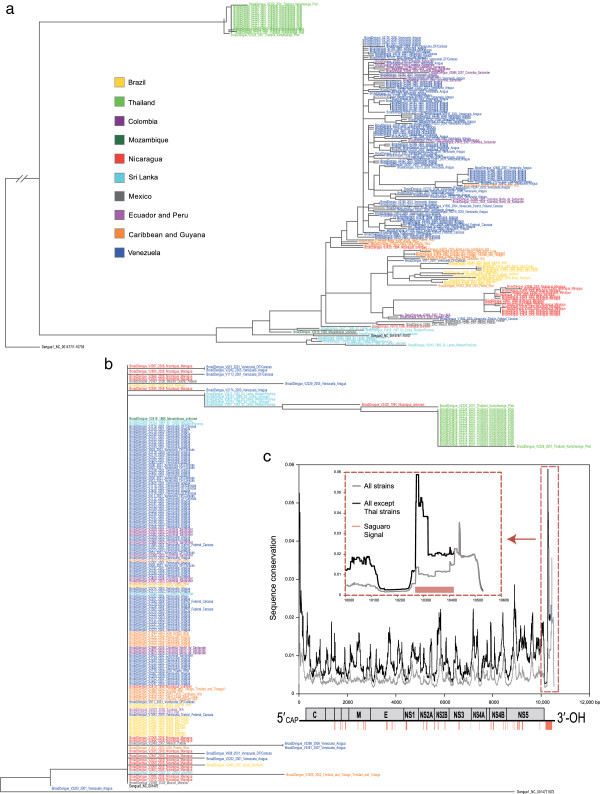
**Lineage-specific conservation in the Dengue virus genome.** (**a**) The phylogeny generated by the regions assigned to the most prevalent cactus was found to closely resemble previous findings [17]. Sequence from Dengue virus serotype 1 was used to root the tree. (**b**) In contrast, the phylogeny based on the most discordant cactus (cactus 5), groups the sequences of all viruses together with the exception of those representing the Thai Dengue virus strains. (**c**) Sequence conservation across the viral genome is plotted for all strains (grey) and after excluding strains from Thailand (black). While the highest level of conservation among all strains is located close to the 3’ end of the virus, the highest conservation peak after excluding the Thai strains coincides with the longest region assigned to cactus 5, indicating high levels of lineage-specific sequence conservation. Shown at the bottom are also all regions modelled by cactus 5 (dark pink).

Phylogenies based on regions covered by cactus 1 through 4 closely resembled previous findings [17], namely that phylogeny followed geographic sampling and year of outbreak (Figure 3a). By contrast, the phylogeny built from the regions identified by cactus 5, which cover 12.6% of the genome in 34 distinct loci, is clearly different (Figure 3b). The sequences from Thailand (light green) show little within-group divergence and form an independent cluster separate from the shorter, collapsed branch lengths of the Central and South American Dengue virus sequences. This group of American Dengue virus strains were collected from recent outbreaks in the early 2000’s and cluster with sequences representing outbreaks in the early 1980’s in Sri Lanka (light blue) and Mozambique (dark green), suggesting shared evolutionary constraint. This phylogeny is consistent with the reported spread of these epidemics from Sri Lanka, through Africa, and into the Caribbean and the Americas in the mid 1980’s [25].

Closer examination of cactus 5 revealed that the longest continuous region was derived from five nucleotide sites spanning 120 bases in the 3’ untranslated region (UTR) of the 3390 amino acid polyprotein Open Reading Frame (ORF). The identification of this signal prompted us to use overlapping sequence windows to test the entire Dengue virus genomes for signs of overall and strain-specific nucleotide conservation. For each nucleotide position *l* in the multiple sequence alignment where at least one genome had a mismatch with another, we determined the smaller number of genomes *n*_*l*_ that differed from each other (analogous to the concept of minor allele frequencies, e.g. if 82 sequences have a C and 99 have a T, then *n* = 82). For each *l*, we then report

$$c_{l}={\left(\overset{l+60}{\underset{i=l-60}{\sum }}n_{i}\right)}^{-1}$$

as a measure of conservation at site *l*.

Figure 3c shows the result graphically, plotting the calculated sequence conservation against the physical length of the viral genome. The value determined using all genome sequences (grey) is illustrated in contrast to that generated by all sequences except those sourced from Thailand (black). While the strongest signal of overall conservation is located close to the 3’ end of the genome (Figure 3c, dotted box, grey), the signal extends in the 5’ direction when the Thai sequences are excluded. This latter signal, masked when overall conservation is computed, is identical to the region identified by cactus 5, and shows signatures of strain-specific conservation in two groups, both the Thailand strains as well as the other strains.

### Genes involved in the immune system leave a distinct trace in five primate genomes

**Figure 4:**
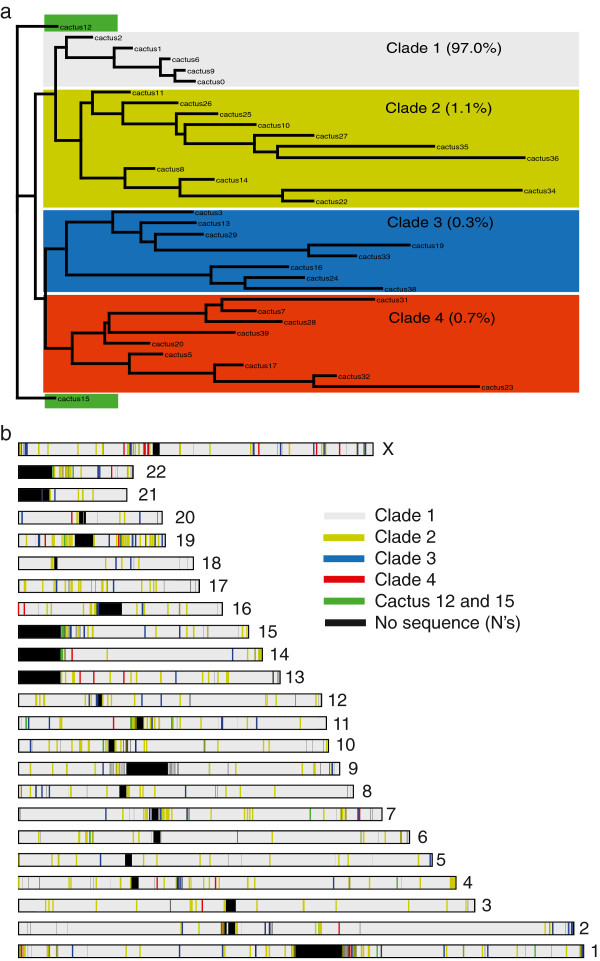
**Cacti computed from five primate genome alignments. (a)** A neighbour joining distance tree groups the cacti into four major clades, with clade 1 (grey) covering 97% of the bases assigned to its genomic segments. The cacti in clades 2–4 (light green, blue and red) and cacti 12 and 15 (dark green) represent the remaining 2.3% and 0.7% of the genome respectively. **(b)** A human-centric ideogram illustrates the distribution of regions assigned to each cacti condensed into clades (1–4) and cacti 12 and 15. While clade 1 describes most of the genome, genomic regions that are better represented by cacti outside of clade 1 are distributed throughout the genome.

We extracted the human, chimpanzee, gorilla, orang-utan and macaque genomes from the Multiz-44 multiple sequence alignments that were used in the analysis of 29 mammalian genomes [26] and imposed filters to mask transposable elements and simple repeats, leaving only positions in which all genomes aligned. After also removing private SNPs, i.e. positions in which only one genome was different and all others were the same, we were left with ~9.47 million positions from which *Saguaro* produced 35 cacti. Figure 4a shows a neighbour joining distance tree of cacti based on their pair-wise Euclidean distances. Rather than exhibiting a star-like shape, which would indicate many different patterns, cacti are placed into four main clades. The top clade (Figure 4a, grey box containing cacti 0–2, 6, 9) captures mostly shared sequence ancestry and covers ~97% of the genome. Phylogenies computed from this dominant clade are similar to each other in terms of branching pattern and length. Notably, cacti representing close to one third of the aligned genomes transposed the relationships between gorilla, chimpanzee and human. This is consistent with previous reports that attribute these regions to the effect of incomplete lineage sorting [27].

Outside of clade 1, there are 30 cacti identifying 747 disjoint regions. Figure 4b shows a human-centric view of the genome-wide distribution of cacti. This ideogram has been coloured to aid visualisation (clade 1, grey; clade 2, yellow; clade 3, blue; clade 4, red; outlier groups cacti 12 and 15 green). Clades 2–4 and the outlier cacti overlap with the introns or exons of 1,159 coding (362) or non-coding (797) genes (Ensembl gene build 64). About 33% (381) of these genes are processed (181) or unprocessed (200) pseudogenes, and an additional 276 non-coding RNAs contain lincRNAs (118), microRNAs (38), and snoRNAs (120). Gene families with more than five members included six synovial sarcoma × genes, 12 UDP glucuronosyltransferases, eight cytochromes, 15 olfactory receptors and two taste receptors, 13 keratins and keratin associated proteins and 14 PRAME family members. Of the 20 zinc finger proteins targeted by this analysis, most were located on chromosome 19 where zinc finger clusters are known to have undergone recent expansions [28]. Interestingly, genes transcribed to form the variable parts of antibodies for Immunoglobolin D (*n* = 20) and Immunoglobolin V (*n* = 36) figured prominently, as did Immunoglobolin V pseudogenes (*n* = 37). Other immunology-related findings included immunoglobulin lambda-like polypeptide 1 (*IGLL1*), immunoglobulin superfamily member 3 (*IGSF3*), 13 HLA genes located in the major histocompatibility complex, eight interferon alpha genes, and the interferon gamma-inducible protein 16 (*IFI16*). GO-term analysis using Ingenuity PA (http://www.ingenuity.com/) recovered additional genes involved in inflammatory/immune response (*p* = 0.017). Among the top ranked genes (in terms of *p*-value) were *APOL1*, *APOL3*, *APP*, *CASP1*, *CASP5*, *CEACAM1*, *CR1*, *CROCC*, *CSF2RB*, *CXCL6*, *E2F2*, *GBA*, *GGT5*, *KIR3DL1*, *MYLK*, *NBN*, *NOS2*, *PARP4*, *RABGEF1*, T*NFRSF10B*, *TNFRSF14*, *ULBP2*, and *XBP1*.

## Conclusions

While an HMM can accurately segment a stream of features into various patterns, it lacks the ability to a priori hypothesise what these patterns are. Conversely, a SOM will cluster signals into distinct patterns automatically, albeit without a spatial component to allow for determination of signal patterning. Through the interleaved application of both algorithms, *Saguaro* allows the strengths of each approach to be exploited. *Saguaro*’s features are nucleotide positions in which genomes are compared, and its patterns, *cacti*, are matrices that robustly model the phylogenetic relationships between organisms.

We demonstrated that *Saguaro* was successfully able to process two data sets at opposite ends of the spectrum; one with many sequences of short lengths, the other with few but complex and large sequences, and in each case identify local phylogenetic branching patterns that differed from the phylogeny as a whole. In 181 strains of Dengue virus serotype 3 [17], we find a 120 nucleotide long region in the 3’UTR (Figure 3c) previously described to contain functional RNA loop structures [29]. This region appears to be under constraint in a lineage-specific manner, and does not appear as a strong signal when looking for conservation across all strains. Moreover, *Saguaro* found this region when only examining the pattern of five informative nucleotide sites, ignorant to the invariant nucleotide positions in between. In primates, *Saguaro* finds four clades of cacti, including one representing the phylogenetic background of the species (Figure 4, clade 1 representing 97% of the genome). In that major clade, one third of the sequence resided in slightly shuffled phylogenies, which, in keeping with a similar fraction previously reported for the gorilla genome, we attribute to incomplete lineage sorting [27]. In addition, *Saguaro* assigns 3% of the aligned genomes to cacti that reside outside of clade 1. Many of these regions overlap with non-coding and coding genes, such as olfactory and taste receptors, as well as zinc finger proteins that could be involved in the regulation of a number of cellular processes. However the strongest signal was associated with inflammatory and immune response genes, sequences that are also known to be highly variable in human populations [30]. Interestingly, a large number of pseudogenes were also identified. Assuming that these are the product of duplications, this finding would not be surprising, as pseudogenisation is considered a common outcome of such duplication events. We note that *Saguaro* is agnostic to the underlying mechanisms that give rise to its cacti, and that if the data contains systematic artefacts, it will likely report them as signals represented by their own cacti. This is a particularly relevant caveat in the case of genomic regions that are inherently difficult to assemble correctly from Whole Genome Shotgun reads, and some of these regions identified in our study of primates, e.g. the major histocompatibility complex (MHC), fall into this category. We thus manually inspected a number of additional regions assigned to the same cactus as the MHC, and found that the majority showed no obvious reasons as to why those should contain assembly errors.

An organism’s ability to adapt and thrive in a given environment is a product of many complex genetic interactions. We expect that the fields of genomics and population genetics will be able to exploit the novel combination of a Hidden Markov Model and a Neural Network contained within *Saguaro* to investigate existing and future data sets with a fresh perspective. The examination of phylogenies without the constraint of a priori assumptions may reveal previously hidden relationships, such as those between hosts and their pathogens, or offer insight into previously unknown biological drives.

### Availability and requirements

Project name: SaguaroGW

Project web site: http://saguarogw.sourceforge.net/

Operating systems: GNU/Linux

Programming language: C++ (Saguaro), perl (data simulation)

Compiler: gcc 4.6.3

Minimum RAM: 4GB, 64+GB recommended

License: free to all users under the LGPL license

## Competing interests

The authors declare that they have no competing interests.

## Authors’ contributions

NZ, PR, NV, EM, and MGG implemented the software. PR provided the mathematical formulation. JRSM, PJ, HL, MPH, FdP, KLT, and MGG designed the dengue and primate experiments. NV designed and performed the simulations. PJ provided the biological interpretation for the dengue results. HL provided phylogenies. All authors wrote the manuscript and designed the figures. All authors read and approved the final manuscript.
